# PUMA and NF-kB Are Cell Signaling Predictors of Reovirus Oncolysis of Breast Cancer

**DOI:** 10.1371/journal.pone.0168233

**Published:** 2017-01-18

**Authors:** Chandini Thirukkumaran, Zhong-Qiao Shi, Ponnampalam Thirukkumaran, Joanne Luider, Karen Kopciuk, Jason Spurrell, Kate Elzinga, Don Morris

**Affiliations:** 1 Translation Research Laboratories, Tom Baker Cancer Centre, Calgary, Alberta, Canada; 2 Alberta Provincial Laboratories, Hospital Dr N.W., Calgary, Alberta, Canada; 3 Calgary Laboratory Services, Foothills Medical Centre, Calgary, Alberta, Canada; 4 Dept of Cancer Epidemiology and Prevention Research, Cancer Control Alberta, AHS, Holy Cross Centre, SW, Calgary, Alberta, Canada; 5 Department of Medicine and Oncology, University of Calgary, Tom Baker Cancer Centre, Calgary, Alberta, Canada; Memorial University of Newfoundland, CANADA

## Abstract

**Background and purpose:**

Reovirus is a ubiquitous RNA virus that exploits aberrant signaling pathways for its replication. The oncolytic potential of reovirus against numerous cancers under pre-clinical/clinical conditions has been documented by us and others. Despite its proven clinical activity, the underlying mechanisms of reovirus oncolysis is still not well elucidated. If reovirus therapy is to be optimized for cancer, including breast cancer patients, it is imperative to understand the mechanisms of reovirus oncolysis, especially in treatment of resistant tumour.

**Experimental approach and results:**

In the present study global gene expression profiling was utilized as a preliminary roadmap to tease-out pivotal molecules involved in reovirus induced apoptosis in breast cancer. Reovirus treated HTB133 and MCF7 breast cancer cells revealed transcriptional alteration of a defined subset of apoptotic genes and members of the nuclear factor-kappa B (NF-kB) family and p53 upregulated modulator of apoptosis (PUMA) were prominent. Since NF-kB can paradoxically suppress or promote apoptosis in cancer, the significance of NF-kB in reovirus oncolysis of breast cancer was investigated. Real time PCR analysis indicated a 2.9–4.3 fold increase in NF-kB p65 message levels following reovirus infection of MCF7 and HTB133, respectively. Nuclear translocation of NF-kB p65 protein was also dramatically augmented post reovirus treatment and correlated with enhanced DNA binding. Pharmacologic inhibition of NF-kB lead to oncolytic protection and significant down regulation of PUMA message levels. PUMA down regulation using siRNA suppressed reovirus oncolysis via significantly repressed apoptosis in p53 mutant HTB133 cells.

**Conclusions:**

This study demonstrates for the first time that a prominent pathway of reovirus oncolysis of breast cancer is mediated through NF-kB and that PUMA upregulation is dependent on NF-kB activation. These findings represent potential therapeutic indicators of reovirus treatment in future clinical trials.

## Introduction

Cancer remains a major health burden worldwide and amongst cancer incidence breast cancer (BrCa) ranks first among female malignancies and is second only to lung cancer in cancer deaths [1,2]. Despite the advancements in diagnosis and treatment of BrCa, globally there is an upward trend in female mortality due to BrCa begging for better treatment options for this malignancy [1–5].

Current treatments for advanced BrCa are limited by lack of efficacy, cellular resistance and toxicity. Dose-escalation, combination and targeted therapies designed to overcome resistance and enhance efficacy are limited by efficacy and/or a narrow therapeutic index. Oncolytic viruses represent a group of novel therapeutics that appear to have an extensive spectrum of anti cancer activity with minimal human toxicity.

One such virus, reovirus is a common environmental double stranded virus which has demonstrated negligible pathogenicity in humans [6]. Initial studies with regards to the underlying mechanisms of reovirus preferential toxicity in transformed/malignant cells has been shown to be due to activated oncogenic cell signaling and not at the cellular receptor status [7, 8]. Further to this, reovirus oncolysis has been shown to be mediated via the Ras/RalGEF/p38 pathway in an NIH 3T3 model system [9]. The importance of proteolytic disassembly of the reovirus capsid and the prevalence of proteases that play a role in successful reovirus oncolysis has also been shown by other others [10–12]. More recently Kelly et al. [13] has demonstrated reovirus sensitivity to be correlated to over expression of reovirus internalization receptor Junctional Adhesion Molecule-A (JAM-A) in multiple myeloma. Thus it appears that multiple factors contribute to successful reovirus entry, dissemination and usurping of the host cell signaling cascade that culminate in efficacious oncolysis.

Our group and others have documented reovirus’s extensive preclinical as well as clinical efficacy against numerous histologies, [14–20]. A randomized phase II/III clinical trial involving reovirus with head and neck cancers has recently been completed. Although the results of this trial have not been published yet, early observations have reported to be encouraging [21]. In addition, there has been four recently completed randomized phase II clinical trials in breast, non -small cell lung cancer, colorectal and prostate cancer undertaken by the Canadian Clinical Trial Group (CCTG) that will be reported in 2017.

Despite this progress, knowledge about precise mechanisms of cell death induced by reovirus in different tumor types is lacking. If reovirus therapy is to be optimized for patients, it is imperative to identify the mechanisms of virus mediated cell death of various cancers. Preliminary evidence from our laboratory and others suggest that reovirus oncolysis of cancer cells is mediated mainly via apoptotic mechanisms [17, 22–24]. The transcription factor nuclear factor- kappa B (NF-kB) has been shown to be an important molecule in apoptosis induction by reovirus strain type 3 Abney [3TA] in the cervical carcinoma cell line Hela [25]. However, in a human embryonic kidney cell line [HEK 239] reovirus induced NF-kB has been shown to act through apoptosis at early time points (4 hours post infection, hpi) and to suppress apoptosis at later time points (12 hpi) [26]. In a study conducted in ovarian, breast and lung cancer cell lines, reovirus infection has been shown to result in sensitizing these cells to TRAIL (TNF Related Apoptosis Inducing Ligand) and to act synergistically with TRAIL to enhance apoptosis [27]. In contrast, in colorectal cancer cell lines a direct interaction between reovirus and TRAIL has not been observed [28]. In colorectal cells, reovirus mediated apoptosis has been shown to be dependent on the *ras* mutation status of the cell and not on reovirus replication capabilities [28]. Thus, it appears that reovirus induced apoptotic signaling pathways in cancer cells are diverse and histology specific. This is not surprising as the malignant tumour phenotype is the culmination of multiple mutations that eventually lead to diverse aberrant signaling pathways that oncolytic viruses exploit for their replication.

To date, no comprehensive study has been undertaken to evaluate reovirus oncolysis mechanisms in BrCa. This knowledge could help in the design of novel approaches in maximizing reovirus’s therapeutic efficacy especially in therapy resistant disease. In the present study we have identified key signaling molecules of reovirus oncolysis of BrCa and demonstrate for the first time that reovirus treatment of BrCa leads to significant augmentation of NF-kB gene transcription, nuclear localization, and upregulation of p53 upregulated modulator of apoptosis (PUMA). These events culminate in apoptosis irrespective of the p53 status of BrCa cells. Thus, it appears that both NF-kB and PUMA could serve as predictive indicators of BrCa sensitivity to reovirus.

## Materials and Methods

### Cell lines

Established BrCa cell lines HTB133, HTB 132, MCF7 and HTB30 were obtained from the American Type Culture Collection (ATCC, Rockville, MD). HTB and MCF7 cells are estrogen receptor (ER) positive whereas HTB132 and HTB30 cells are ER negative [29]. All cells were grown in DMEM media (Gibco BRL, Burlington, Ontario) containing 10% fetal bovine serum (FBS), supplemented with sodium pyruvate and HEPES.

### Reovirus apoptotic assays

Reovirus serotype 3 (strain Dearing) was propagated in L929 cells and purified as previously described [30]. MCF7 and HTB 133 cells were infected with either live virus (LV) or UV inactivated virus (DV) (40 MOI), or untreated (NV) for 0, 2, 3, 4, and 6 or 7 days. 1mL of the harvested cell suspension (~ 1 X 10^6^) was centrifuged at 450x g for 1 minute and the cell pellets were washed in PBS twice before subsequent staining with propidium iodide (PI)/RNase A, annexin-V-FITC/7AAD or Apo 2.7 as previously described [17]. Flow cytometric analysis was performed in a Bekman Coulter XL flow cytometer (Beckman Coulter, Mississauga, Ontario, Canada) using FCS EXPRESS software. To assess caspase activity, BrCa cells were infected with LV or DV (40 MOI) or NV. Cell monolayers were trypsinized at 72 hpi. and 1 x 10^6^ cells were used to measure *in situ* caspase activity using Caspa Tag™ caspase- 3/7 (LETD) caspase assay kits (Intergen Company, NY) as per manufacture’s protocols. For bi-colour analysis, 20 μl of 7-AAD was added to samples and cell death and caspase activity was monitored via flow cytometry.

### Microarray analysis

HTB 133 and MCF7 breast cell lines were grown to 75% confluency and infected with LV or DV for 12 and 24 h and total RNA was extracted using trizol (Invitrogen). Quantified RNA was subjected to DNase treatment and the integrity of the purified RNA was confirmed following gel analysis. 25 μg of RNA was reverse transcribed to cDNA and labeled with Cy3 and Cy5 via the dye coupling method. Labeled cDNA was column purified and hybridized onto 14 k oligo gene chips (Southern Alberta Microarray Facility, Canada), and incubated overnight. Following washing and scanning, the signal intensity was quantified and analyzed using the “GeneTraffic” software [31]. Key apoptotic signaling molecules that were up/down regulated ≥2 fold were identified. Details of microarray data could be accessed at the GEO database (accession number- GSE69813).

### Electrophoretic mobility shift assays (EMSA)

BrCa cells were either treated with 20 ng/ml of TNF-α or infected with reovirus (40 MOI). Nuclear and cytosolic proteins were prepared essentially as described by Connolly et al. [25]. EMSAs were carried out utilizing an infrared dye 700 labeled oligonucleotide (IR oligo) consisting the NF-kB consensus binding sequence (Odyssey, Li-Cor Biosciences, Lincoln, Nebraska, USA). The binding reaction consisted of 1x binding buffer (10mM Tris, 50 mM KCl, 1 mM DTT, pH 7.5), sterile water, 1 μg of poly[dI-dC] (Amersham Pharmacia Biotech, USA) in 10 mM Tris and 1mM EDTA pH 7.5, 2.5 mM DTT/0.25% Tween 20, and 1 μl of NF-kB IR oligo. Five μg of nuclear protein was added to the mixture and incubated at room temperature for 30 min in darkness. For supershift analysis, the above mixture was incubated with 5 μg of NF-kB antibody p50 (SC-7178X Santa Cruz) or p65 (SC-109X, Santa Cruz) for an additional 30 minutes. The nuclear protein-DNA complexes were separated in native 4% polyacrylamide gels and scanned in a Li-Cor Odyssey infrared imaging system (Lincoln, Nebraska).

### Western blot analysis

Western blotting was carried out as described previously [17]. Blots were probed with rabbit anti-NF-kB p65 (SC-109, Santa Cruz Biotechnology, Santa Cruz, CA, USA), or rabbit anti-human Puma (ABCam Inc., Cambridge, MA, USA). Mouse monoclonal anti-actin (MAB 1501) and moue anti-histone loading controls were from Chemicon International.

### Pharmacological inhibition of NF-kB and cytotoxicity assays

For pharmacologic inhibition of NF-kB, we chose two compounds, N-Acetyl-Leu-Leu-Norleu-al (ALLN) and Caffeic acid phenethyl ester (CAPE) (Calbiochem, San Diego, CA, USA). Both these compounds are reported to inhibit NF-kB but their modes of inhibition appear to be different. ALLN is a general proteasome inhibitor [32] where it can prevent the degradation of IkB at the proteasome and thus prevent the release of NF-kB [32] whereas CAPE prevents the translocation of NF-kB and has no effects on IkB degradation [33]. We thus considered it important to compare the effects of these 2 compounds on reovirus mediated NF-kB activity in BrCa cells. Initial dose escalation studies on cell viability conducted with CAPE and ALLN indicated the optimal doses that were non- toxic to breast cells to be 10 μM for ALLN and 20 μM for CAPE (see S1 and S2 Figs). To assess pharmacological inhibition of NF-kB, MCF7 or HTB133 cells were pre-incubated with either CAPE (20 μM) or ALLN (10μM) for 3h before infection with reovirus at 40 MOI. Cell viability was assessed via the WST assay [34].

### Quantitative real time RT-PCR (qRT-PCR)

RNA was extracted from HTB 133, HTB132 and MCF7 cells that were treated with LV or DV (40 MOI) at 12, 24 and 48 hpi using Trizol. For pharmacologic inhibition of NF-kB, cells were treated with CAPE (20 μM) or ALLN (10 μM) for 3 hours prior to infection with LV or DV for 24h. Total RNA was reverse transcribed using superscript III reverse transcriptase and N6 random primer (Invitrogen) and qPCR was performed using TaqMan gene expression master mix (Applied Biosystems). Each sample was thermocycled in triplicate using PUMA (Hs00248057_ml) and NF-kB (Hs00153294_ml) specific TaqMan assays containing primers and probes (Applied Biosystems). 18s rRNA (Applied Biosystems, 4319413E) was used as the endogenous control to normalize the concentration variations between the samples. Prior to conducting real time PCR assays the best house—keeping control gene that could be used in reovirus assays was verified using the TaqMan Human Endogenous Control Plate Cat# 4309199, Applied Biosystems. As shown in S1 Table we confirmed that the best gene to be used in reovirus assays to be 18S which gives the minimal differences in CT values between treatments and controls. Data analysis was carried out using 7500 software v 2.0.1.

### Silencing studies using small interfering RNAs (siRNAs)

siRNA silencing of the PUMA gene was confirmed via two independent experiments utilizing siRNA from 2 different sources: PUMA, (*BBRC)*, (Cat. #16104) and Control (Cat. # 4613) (Ambion Inc., Austin, Texas) and ON-TARGETplus SMARTpool siRNA against PUMA (L004380-00) and GAPDH (D-001140-01-05) (Dharmacon RNA Technologies, Chicago, IL.). MCF-7 and HTB133 (1.2x10^5^ cells/well) were seeded in 1ml of DMEM + 10% FBS in 12-well plates to achieve 50–60% confluency in 24 hours. Transfection was performed in serum free medium with HiPerfect (Qiagen) or Metafectene Pro reagent (Biontex Laboratories GmbH, Munich, Germany). The siRNA-lipid complexes (2.5μl: 0.5μg) were incubated at room temperature for 20 minutes and added to the cells. Following incubation at 37°C for 3 hours the medium was supplemented with 10% serum. Total cellular protein was extracted at 24, 48, 72 hours and PUMA down regulation was monitored via western blotting. Transfection conditions were initially optimized using GAPDH siRNA.

To verify effects of PUMA down regulation on reovirus induced apoptosis, MCF7 and HTB133 cells were transfected with 50nM Puma siRNA for 24 hours and then infected with reovirus (40 MOI). Samples were collected at 24 and 48 hours. Reovirus oncolytic protection was monitored via trypan blue incorporation and apoptosis was determined by flow cytometry using annexin-V–FITC/PI staining.

### Reovirus progeny assays

In order to investigate the effects of CAPE and ALLN on reovirus progeny production, breast cancer cells were grown in six-well plates and infected with either 40 MOI of reovirus or reovirus+CAPE (20μM) or reovirus+ALLN (10μM). Plates were incubated for varying time points up to 48 hours and frozen at -80C. Following three freeze-thaw cycles of the frozen cells, the supernatant was subjected to plaque titration of L929 cells. The experiment was repeated two times for each cell line.

### Statistical methods

Graphical and tabular data summaries were used to describe all experimental results. Statistical tests of hypotheses were carried out for two samples using *t*-test statistics that assumed unequal variances and for more than two groups using one way analysis of variance (ANOVA). Dunnett’s test was used for comparing multiple inhibitors to the no inhibitor control. All tests adopted two-sided alternatives and statistical significance level was 0.05. The *R* statistical program was used for all analyses [35].

## Results

### Reovirus oncolysis of BrCa occurs via apoptosis

**Fig 1:**
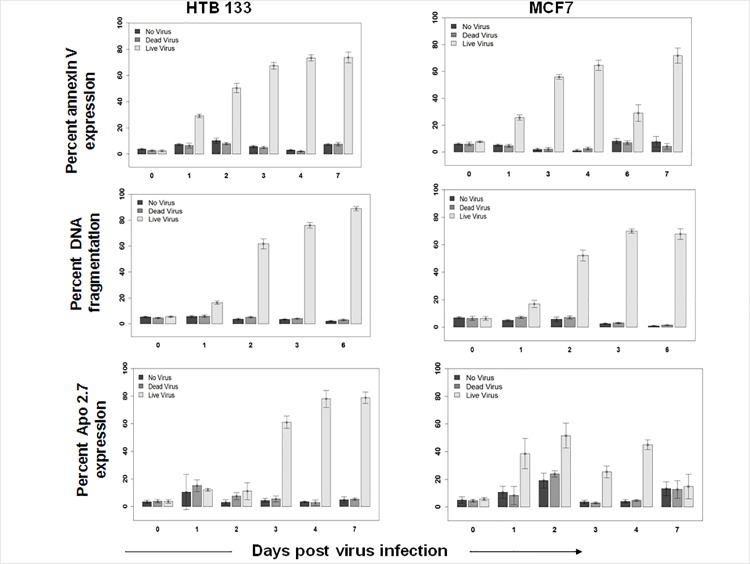
Reovirus induces apoptosis in BrCa cell lines. HTB133 and MCF7 cells were harvested at indicated time points following treatment with no virus (NV), dead virus (DV), or live virus (LV). Apoptosis was measured using flow cytometry via Annexin V binding, DNA fragmentation and Apo 2.7 expression. (N = 3, ± SD).

Evidence that reovirus exerts its oncolytic effect through apoptosis was confirmed via flow cytometry using 3 different markers i.e., phosphatidyl serine expression (Annexin V binding), DNA fragmentation (PI incorporation), and mitochondrial dysfunction (Apo 2.7 staining) in two breast cancer cell lines initially. As shown in Fig 1, a dramatic increase in Annexin V staining (60–70%) was seen between 1–7 days of LV but not DV or NV treatments of HTB133 and MCF7 cells. Similarly, DNA fragmentation reached a maximum at 6 or 7 days post LV infection. The altered expression of Apo 2.7 maximized at 2 days (52%) in MCF7 and at 7 days (79%) in HTB133 cells following LV infection suggesting the involvement of the intrinsic apoptotic pathway.

**Fig 2:**
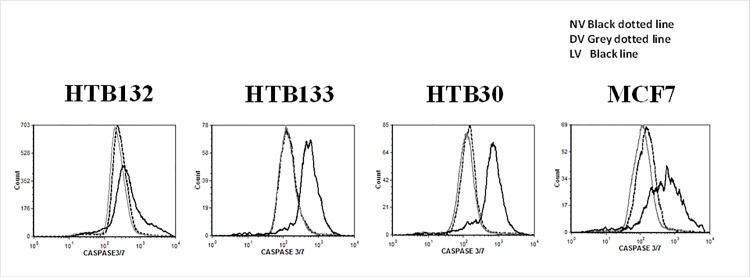
Caspase 3/7 is activated during reovirus induced apoptosis of BrCa cells. BrCa cells were infected with No virus (NV), LV and DV at a MOI of 40 PFU/cell, or untreated (NV). Cells were harvested at 72h and *in situ* active caspase 3/7 activity was measured using flow cytometry.

In order to confirm that terminal caspases 3/7 is activated in reovrius mediated apoptotic process, we verified its activity in a panel of breast cancer cell lines using flow cyotmetry. As shown in Fig 2, active caspase 3/7 was makedly increased in all LV treated BrCa cells in comparion to cells that were untreated or DV treated confirming reovirus oncolysis is mediated via apoptosis.

### Gene expression profiling

Global gene expression profiling was next utilized as a preliminary roadmap to tease-out pivotal molecules involved in reovirus induced apoptosis in BrCa. Reovirus infection at 24h resulted in expression changes (defined as over 2 fold) of 331 and 332 genes in MCF7 and HTB133 BrCa cells respectively. Of these, a subset of genes related to apoptosis were categorized as depicted in Table 1.

**Table 1:** Transcriptional upregulation of apoptotic genes in HTB 133 and MCF breast cancer cells post reovirus infection*.

	Gene	Gene ID	Cell Line				
			HTB 133		MCF7		
			Time Post Reovirus Infection				
			12h	24h		12h	24h
Apoptosis related Transcription factors	Ik-B- (NF*k*B Inhibitor)	4792	13.64	56.5		11.71	15
	NF-kB p100/49 Precursor of p52	4791	3.48	7.21		2.04	2.33
	NF-kB p65	396027	5.42	4.03		1.83	1.70
	NF-kB p105 Precursor of p50	81736	2.11	2.53		1.6	1.3
	STAT2	6773	NA**	4.34		NA	1.6
	STAT 5A	574129	4.86	3.23		1.67	1.5
	Apoptosis Antagonizing T.F	26574	NA	NA		1.75	1.53
	SOX 4	6659	2.36	2.06		NA	NA
Death receptor associated genes	TNF-alpha induced protein 3	7128	12.5	33		8.4	12.73
	TRAIL receptor 2	8795	4.53	3.05		2.02	NA
	TNFR member 6	355	4.35	2.25		NA	NA
	TNF member 1		2.65	2.06		NA	NA
	FAS associated factor	11124	2.0	1.73		NA	NA
	Apoptosis inhibitor 2	330	NA	3.43		NA	1.5
P53 associated genes	PUMA (P53 up regulated modulator of apoptosis)	27113	27.66	8.82		2.43	2.35
	P53 induced gene	7157	17.15	NA		3.55	2.5
	P53 binding protein MDM4	4194	2.2	2.78		1.87	2.64
	P53 binding protein MDM2	4193	2.2	3.41		2.0	3.4
Caspase related genes	Caspase 4	837	NA	5.62		1.31	NA
	Caspase 5	838	1.3	3.41		1.46	1.41
	Caspase 8 and FADD-like apoptosis regulator	8837	1.91	2.36		1.75	1.34

*Altered Expression (fold change) in live virus versus dead virus treated cells,

**NA-Results not available.

#### [i] Altered expression of apoptotic genes in relation to transcription factors

Members of the Rel/NF-kB transcription family were among the most significantly upregulated genes in reovirus treated BrCa. Of the 5 related NF-kB family members i.e., p50 (NF-kB1), p52 (NF-kB2), Rel A (p65), Rel B and c-Rel [36], alterations in expression of three NF-kB family genes was seen at 12 and 24 hpi in both HTB 133 and MCF7 cells. Although the de-novo synthesis of NF-kB is usually uncommon following external stimuli, our results imply that reovirus treatment leads to transcriptional upregulation of p65, P100 (precursor of p52) and P105 (precursor of p50). Reovirus treatment also resulted in 2.06–4.86 fold increase in STAT2, STAT5A and SOX 4 expression in HTB 133 cells.

#### (ii) Altered expression of death receptor [DR] associated genes

As shown in Table 1 reovirus treatment of BrCa cells lead to the upregulation of a variety of DRs including TRAILR2, Fas and TNFR associated genes. Similarly, DR associated proteins/ligand expression was also noted. An 8.4–12.5 fold increase in expression of TNF-α induced protein 3 (TNF-αIP) was detected in both cell lines and a 2.06–2.65 fold increase in TNF-β expression was seen in HTB 133. Taken together, these results suggest multiple apoptotic signaling pathways ensue through a variety of DR activation following reovirus infection of BrCa. The functional involvement of individual DRs will need to be assessed experimentally to confirm the contribution of these DR upregulation in reovirus oncolysis.

#### (iii) Altered expression of p53 associated and caspase related genes

Among the p53 associated genes, PUMA was highly upregulated (8.22–27.66) in p53 mutant HTB 133 cells whereas this was moderately upregulated (2.43) in the p53 proficient MCF7 cells. “P53 induced gene” expression was 17 fold increased in HTB133 at 12hpi. P53 binding proteins MDAM2 and MDAM4 expression was moderately (2.0–3.4) increased in both cell lines. Interestingly only HTB 133 expressed moderate increases (2.36–5.62 fold) in Caspase 4, 5 and Caspase 8 and FADD like apoptosis regulator.

### Reovirus upregulates NF-kB activity in BrCa

**Fig 3:**
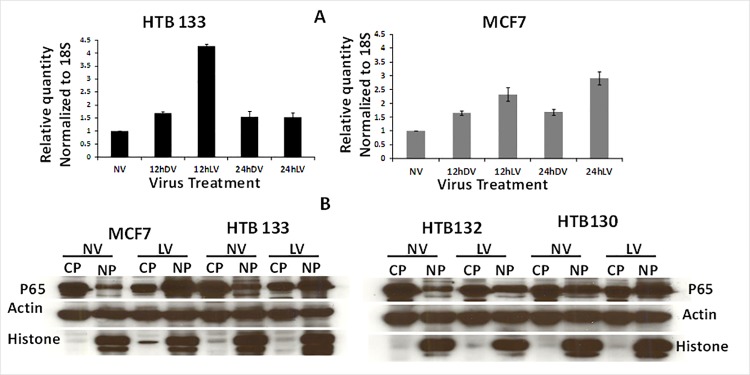
Reovirus upregulates NF-kB activity in BrCa cells. (A) NF-kB p65 mRNA expression is induced in reovirus infected BrCa cells. HTB133 and MCF7 cells were infected with 40 MOI of LV or DV for 12 and 24 hours. Total RNA extracted was assessed for NF-kB p65 and 18S gene expression by qPCR. Bars represent fold increase over untreated controls. (N = 2, ± SD). (B) Reovirus up regulates nuclear translocation of NF-kB in BrCa cells. BrCa cells were incubated with 40 MOI of reovirus for 12 hours and nuclear and cyto solic extracts were prepared. NV or LV treated proteins were subjected to SDS/PAGE and blotted with anti NF-kBp65, actin and histone antibody.

Since NF-kB and PUMA were the most significantly upregulated apoptotic molecules in gene expression arrays, we chose to validate these results via qPCR and western blotting. As depicted in Fig 3A, our real—time qPCR data supported those of gene expression profiling. Maximum fold changes of NF-kB p65 message levels with LV treatment were detected to be 4.3 for HTB 133 at 12 hpi whereas for MCF7 this was 2.9 at 24hpi indicating *de novo* synthesis of NF-kB. Reovirus infection also led to augmented nuclear translocation of NF-kB in all 4 BrCa cell lines (Fig 3B). Significant increases in nuclear localized NF-kB was seen with LV treatment in comparison to NV treatment.

**Fig 4:**
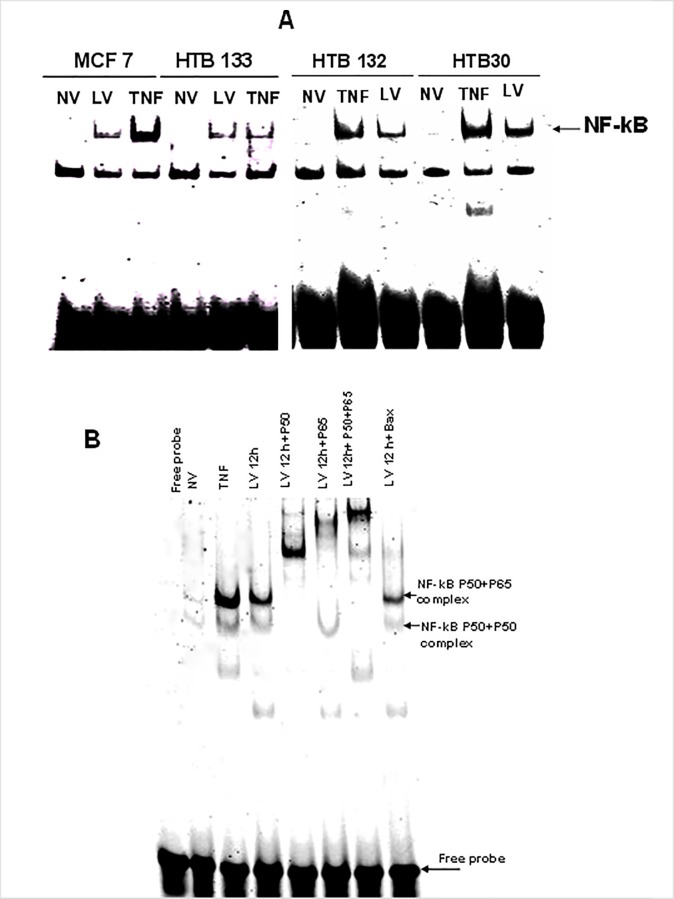
Functional aptitude of reovirus activated NK-kB in BrCa. (A) Reovirus induces NF-kB DNA binding in BrCa cells. BrCa cells were treated with either NV or LV for 12 hours or TNF (20 ng/ml) and nuclear extracts were prepared. 5 micro grams of nuclear proteins was incubated with an IR-dye labeled oligonucleotide consisting of the NF-kB consensus binding sequence and resolved by acrylamide gel electrophoresis and scanned in a LI-COR gel scanner. (B) NF-kB super shift complex is made up of p50 and p65. Nuclear proteins were incubated with 2 μl of super shift antibodies directed against NF-kB p50 or p65 before incubation with the NF-kB p65 probe. The mixtures were resolved in acrylamide gels and scanned as described under Fig 4A.

We next verified the functional aptitude of this nuclear translocated NF-kB by utilizing EMSA. As depicted in Fig 4A, strong DNA/NF-kB complexes were present in all BrCa cell lines 12 hpi whereas these were absent in the uninfected controls. As shown in Fig 4B, supershift analysis confirmed that the NF-kB/DNA bound complexes comprised both p50 and p65.

**Fig 5:**
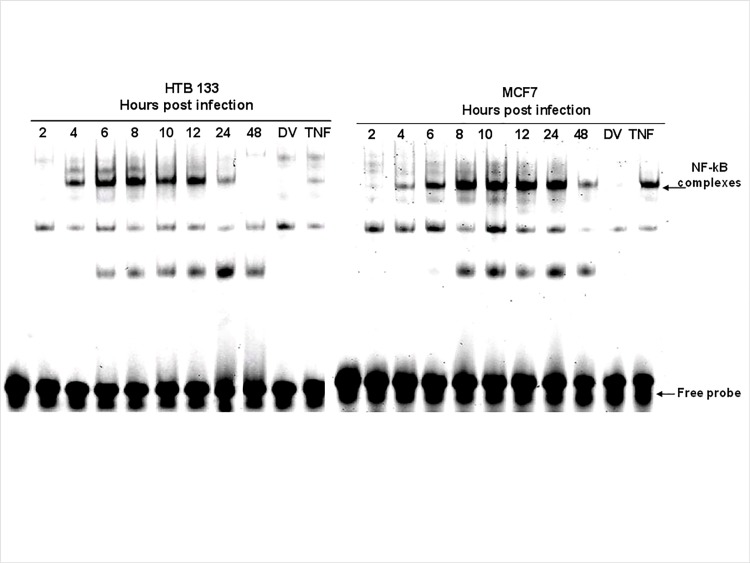
Reovirus induces DNA binding of NF-kB in BrCa cells in a time dependent manner. BrCa cells were treated with either NV or LV or TNF (20 ng/ml) and nuclear extracts were prepared at the times indicated. EMSAs were conducted as previously and scanned.

Having shown that reovirus activates NF-kB nuclear translocation, we next investigated the kinetics of this phenomenon. As depicted in Fig 5, reovirus induced nuclear trafficking of NF-kB was evident 4 hpi and peaked at 12 and 24 hpi. for HTB133 and MCF7 cells. Results of EMSA analysis were confirmed by westerns (data not presented).

### NF-kB acts pro-apoptotically in reovirus oncolysis of BrCa

**Fig 6:**
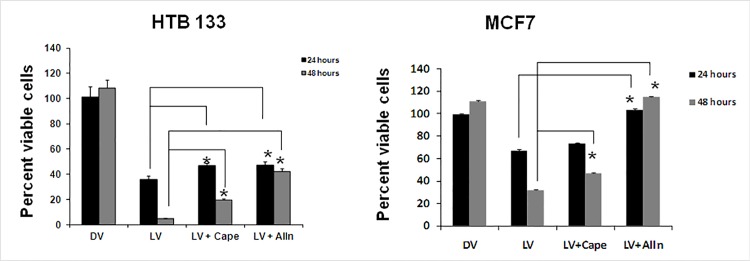
Pharmacologic inhibition of NF-kB leads to suppression of reovirus induced cell death in BrCa cells. MCF7 and HTB 133 cells were pre-incubated with either the NF-kB specific inhibitors CAPE (20 μM), or the proteasome inhibitor ALLN (10 μM), for 3 hours prior to reovirus infection. Cell death was assessed 24, and 48h via the WST assay (N = 5, ± SD). (*), significantly different.

To address the importance of NF-kB in reovirus mediated BrCa oncolysis, we utilized two pharmacologic inhibitors of NF-kB, CAPE and ALLN. As shown in Fig 6 reovirus mediated oncolysis was significantly reversed by ALLN at 24 (*p*-values < 0.02) and 48 hpi (*p*-values < 0.001) in both BrCa cell lines. Significant effects of CAPE were noted at 24hpi for HTB133 and at 48hpi for both cell lines.

### Effects of CAPE and ALLN on reovirus progeny production

As the modes of NF-kB inhibition by CAPE and ALLN are different, we verified the effects of these inhibitors on reovirus replication using viral progeny assays. The two-sample *t*-test was used to determine if adding CAPE or ALLN to reovirus makes a difference in the mean log PFU/ml levels at the three time points (12, 24 and 48 hour) in these small sample groups [37], without adjustment for multiple testing. Plots of the mean changes over time for all three groups enabled descriptive comparisons.

As depicted in S3 Fig, CAPE has no effects on reovirus progeny production at 0, 12 or 48 hours. These results are not surprising as CAPE affects only NF-kB translocation and not proteolysis of IkB [33]. ALLN had no statistically significant effects on reovirus progeny production of MCF7 cells where as in HTB 133 its effects were suppressive at these time points although the plot suggests the difference is diminishing after 24 h. These differences can be explained by the general proteasome inhibitory capacity of ALLN which probably has off target effects [32].

### Reovirus upregulates PUMA gene transcription in BrCa cells

**Fig 7:**
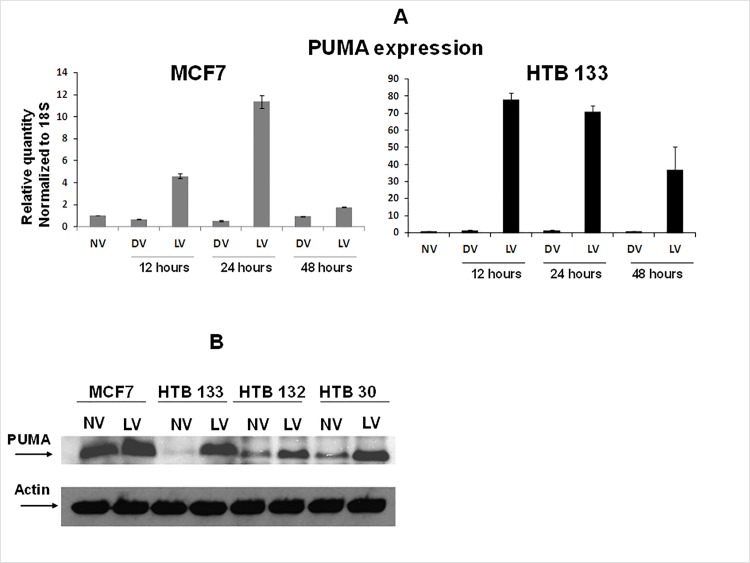
Reovirus infection of BrCa leads to upregulation of PUMA. (A) PUMA mRNA expression is induced in reovirus infected BrCa cell lines. MCF7 and HTB 133 cells were infected with 40 MOI of reovirus for 12, 24 and 48 hours. PUMA gene expression was quantified by real-time PCR. Bars represent fold increase over untreated control cultures (N = 3, ± SD). (B) Reovirus treatment up regulates PUMA protein expression in BrCa cells. BrCa cell lines were incubated with 40 MOI of reovirus for 12 hours and cytosolic extracts were prepared. 50 μg of virus treated (LV) or untreated (NV) proteins were subjected to SDS/PAGE and blotted with anti PUMA antibody and HRP linked secondary antibody.

Since PUMA was another highly upregulated proapoptotic gene seen in our microarray analysis, we verified its amplification by qRT-PCR as well as western blotting. qRT-PCR revealed a 4.6–11.36 fold increases in PUMA mRNA in MCF7 cells with reovirus treatment at 12 and 24 hpi. In HTB 133 cells this increase was significantly augmented leading to >70 fold increases both at 12 and 24 hpi (Fig 7A). The increase in PUMA mRNA also translated to an increase in protein expression as well. As shown in Fig 7B we verified PUMA protein expression in four BrCa cell lines. At 12 hpi reovirus augmented PUMA protein expression in all four BrCa cell lines leading to a 2.94–63.6 fold increase in comparison to the uninfected controls. The highest fold change was observed in HTB133 and the lowest in MCF7 corroborating with the qPCR results.

### Puma gene transcription is regulated by NF-kB

**Fig 8:**
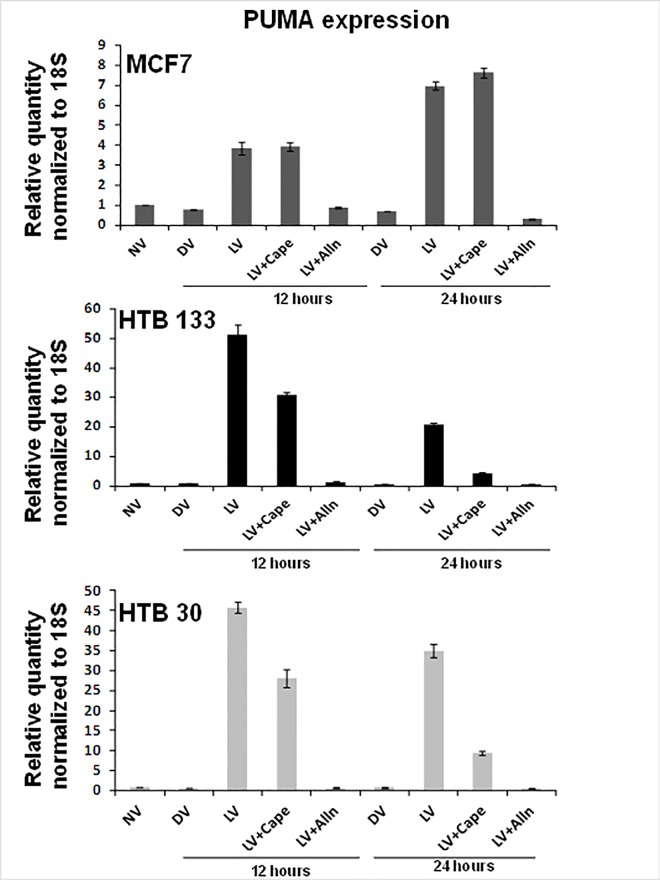
Pharmacologic inhibition of NF-kB down regulates reovirus-induced PUMA gene expression in BrCa cells. Real time PCR was performed on RNA isolated from MCF7, HTB133 and HTB30 cells incubated with either media alone, CAPE (20μM), ALLN (10μM) for 3 hours before incubation with 40 MOI reovirus for 12 hours. Bars represent fold increase over untreated control cultures (N = 3, ± SD).

Having established that NF-kB is a pivotal apoptosis inducing molecule in the present system, we next examined whether PUMA gene transcription is NF-kB dependent. As shown in Fig 8, blockage of I-kB proteolysis with ALLN dramatically down regulated PUMA expression in all 3 cell lines tested. These marked suppressive effects of ALLN on PUMA gene transcription is not surprising as ALLN is a general proteasome inhibitor and could affect the degradation of other off-target proteins as well (32). CAPE, a specific NF-kB inhibitor that inhibits NF-kB translocation and not IkB degradation, was able to down regulate PUMA expression by 17–26 fold in HTB 133 and HTB 30 cells respectively whereas in MCF7 there was no effect.

Since the PUMA promoter is known to consist of 2 NF-kB binding sites [38], these results imply NF-kB to be a transcription factor that regulates PUMA gene expression.

### Molecular inhibition of PUMA leads to modest reovirus oncolysis protection and suppression of apoptosis

**Fig 9:**
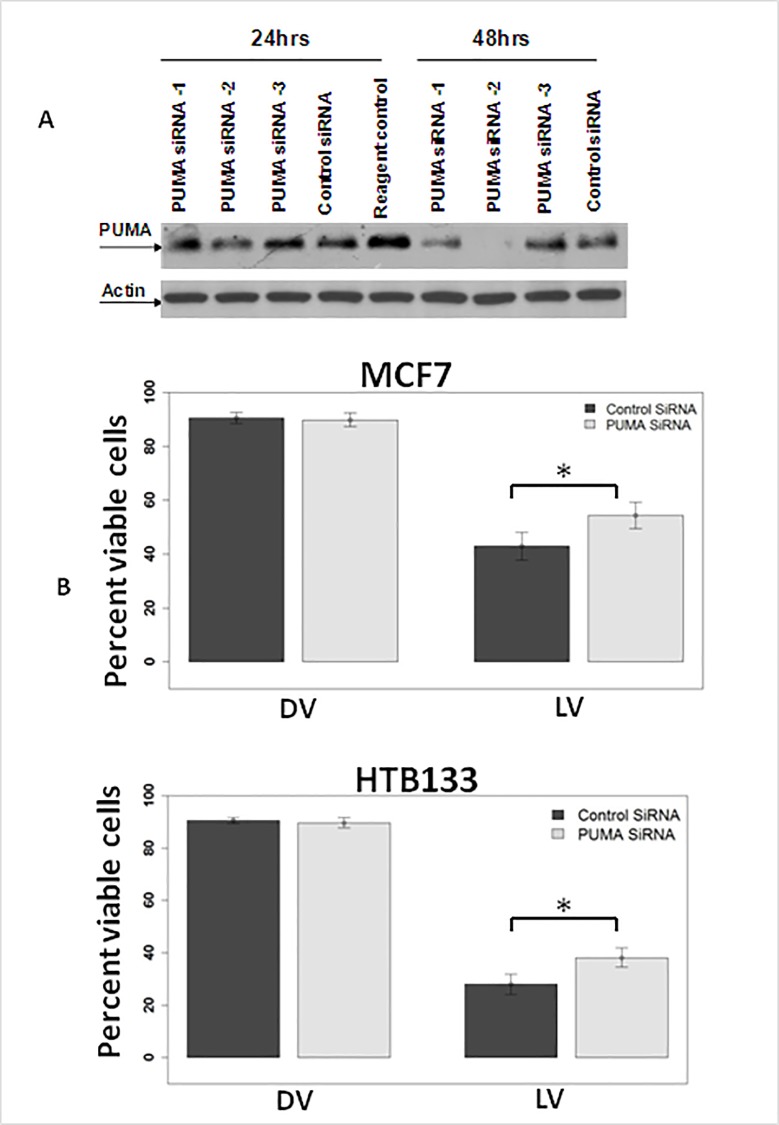
Molecular inhibition of PUMA leads to oncolytic protection. **(A) PUMA down regulation via siRNA leads to suppression of PUMA protein expression.** HTB 133 cells were transfected with siRNA sequences against PUMA (Ambion) and total protein extracted was subjected to western analysis against PUMA and actin antibodies. **(B) Oncolytic protection by PUMA down regulation.** HTB 133 and MCF7 cells were transfected with siRNA sequences #2 against PUMA and infected with live or dead reovirus. Cells death was assayed via trypan blue (N = 3, ± SD).

As many pharmacological inhibitors have off target effects and a better mode of inhibition of a molecule is via a molecular approach, we used siRNA methodology to silence PUMA. Since PUMA was originally described as a P53 upregulated modulator of apoptosis, we used 2 cell lines that were either p53 proficient (MCF7) or p53 mutant (HTB 133) to assess its importance in reovirus mediated apoptosis. Initially HTB 133 cells were transfected with 3 PUMA siRNA constructs from Ambion, Inc. Cellular proteins that were extracted at 24 and 48 hours after were subjected to western blotting. As shown in Fig 9A, the best PUMA protein down regulation was achieved by construct #2. HTB 133 and MCF7 cells were then transfected with PUMA siRNA #2 and infected with reovirus and cell viability was assessed via trypan blue. PUMA down regulation lead to significant (*p* < 0.05) oncolytic protection in both cell lines (Fig 9B).

**Fig 10:**
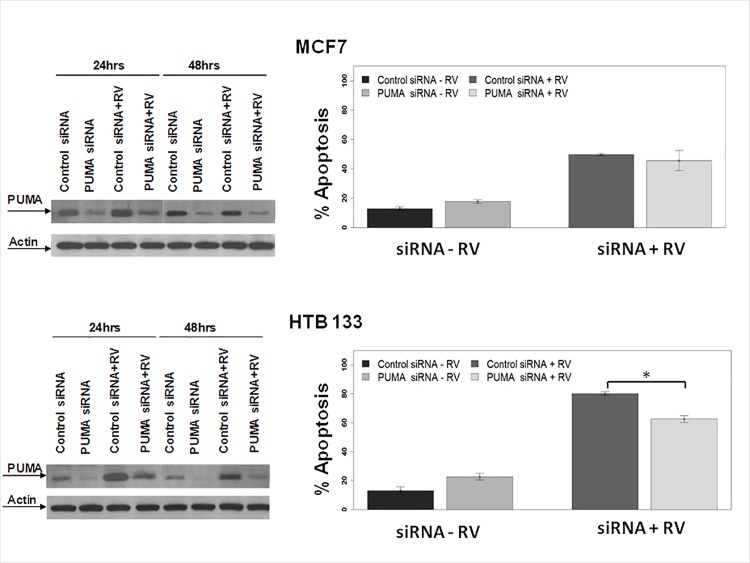
PUMA down regulation leads to modest apoptosis inhibition. MCF7 and HTB 133 cells were transfected with on-target plus siRNA sequences against PUMA or control siRNA and infected with LV. Left panels -Total cellular proteins extracted were subjected to SDS PAGE and blotted against PUMA and actin antibodies. Right panels—HTB 133 and MCF7 cells were transfected with control or PUMA siRNA and infected with LV. Apoptosis was assayed via flow cytometry using annexin V/7AAD, (N = 3, ± SD).

To confirm these findings, we next utilized ontarget plus smart pool siRNA (known to have less off—target effects) and cellular protein was extracted at 24, and 48 hours. Western blot analysis revealed substantial down regulation of PUMA protein at 24 and 48 hours (Fig 10 left panels). To determine whether this down regulation would also lead to apoptosis inhibition, MCF7 and HTB 133 cells were transfected with PUMA siRNA and treated with reovirus for 24 and 48 hours. Apoptosis was assayed via Annexin V binding (Fig 10 right panels). Interestingly, apoptosis was significantly suppressed (*p*< 0.0012) at 48hpi in HTB133 cells that had mutant p53. Although not statistically significant, a mild suppression in apoptosis was also noted in MCF 7 cells that were p53 proficient.

## Discussion

We demonstrate for the first time that reovirus therapy of BrCa leads to the augmentation of NF-kB gene transcription and identify PUMA as a novel, direct NF-kB driven target of reovirus mediated oncolyis. PUMA mRNA and protein were consistently activated in BrCa cells treated with reovirus as revealed by 3 independent lines of evidence: microarray, qRT-PCR and western blotting. PUMA, originally described as a p53 modulated activator of apoptosis [39, 40] and a Bcl-2 interacting partner [41] is now known to be activated in a p53 dependent or independent manner [42]. The activity of PUMA is known to be controlled exclusively by transcription whereas other BH3-only proteins are often activated through multiple mechanisms including posttranslational modifications [42]. Recently it has been established that the distal region of the PUMA promoter contains NF-kB p65 binding sites and in colorectal cancer cells canonical NF-kB p65 signaling via TNF-alpha induction could promote PUMA gene transcription [38]. Consistently in the present study, suppression of reovirus induced NF-kB leads to down regulation of PUMA gene expression, irrespective of the p53 status of BrCa cells. Reovirus upregulated PUMA in 3 functional p-53 deficient cell lines, HTB133, 132 and 30 as well as in MCF7 that was p-53 proficient [wild type p-53]. Thus, reovirus upregulation of PUMA in BrCa is independent of functional p53 and dependent on NF-kB. Further evidence supporting NF-kB to directly activate PUMA in a p53 independent manner comes from a study by Wang et al. [38] where TNF-α stimulation of p53 mutant, p53 wild type or p53 knock out colorectal cancer cells lead to PUMA mRNA and protein upregulation via transcriptional activation of NF-kB.

PUMA down regulation via siRNA lead to modest but statistically significant oncolytic protection in both MCF7 and HTB 133 cells and suppressed apoptosis moderately in p53 mutant HTB 133 cells showing statistical significance when compared to the controls. These findings corroborate with those of Wang et.al [38] where TNF-α treatment of PUMA knockout mice lead to a significant reduction of apoptotic cells in their small intestine and liver, in comparison to wild type mice with identical treatment. PUMA induction by TNF-α was inhibited when the mice were treated with NF-kB inhibitor BAY 11–7082 suggesting that NF-kB can promote TNF- α induced apoptosis by activating PUMA [38]. Although PUMA down regulation weakly repressed apoptosis in p53 proficient MCF7 cells the result was not statistically significant. This discrepancy between MCF7 and HTB 133 could be explained by several factors. Firstly, MCF7 had high basal levels of PUMA protein and the magnitude of PUMA induction by reovirus was comparatively lower in these cells as revealed by qRT-PCR, western and microarray analysis. Further, inhibition of NF-kB by pharmacological means showed dramatic reductions in PUMA message levels in HTB 133 and HTB30 cells whereas in MCF7 cells this suppression was 10 fold less, again probably due to the high basal PUMA levels. Finally, flow cytometric analysis of Apo.2.7 protein expression [indicative of mitochondrial dysfunction] following reovirus treatment was lower in MCF 7 cells [52% compared to 79% in HTB 133 cells]. Thus, it appears that MCF7 represent a different sub class of BrCa histology where the extrinsic apoptotic signaling pathway possibly over rides the intrinsic pathway in response to reovirus treatment.

The ER pathway of these cells likely do not contribute to this discrepancy as both MCF7 and HYB133 are ER positive. Conversely, the magnitude of PUMA down regulation via siRNA in MCF7 may not be sufficient to significantly suppress apoptosis induced by reovirus. The oncolytic protection seen in MCF7 further to PUMA suppression suggests possible cross talk between PUMA and the extrinsic apoptotic pathway.

Recently Pan et al. [43] investigated the relevance of the p53 status of colorectal cancer cells lines in reovirus mediated apoptosis. Utilizing a p53^+/+^ HCT116 colorectal cancer cell line and its p53 ^-/-^ (null) derivative, they demonstrated that reovirus infection leads to the upregulation of p53 target genes PUMA, NOXA and p21 in both cell types. However, when these cells were treated with Nutlin3a [an MDAM2 repressor] and reovirus, p53 accumulation lead to enhanced apoptosis in p53 WT cells but not in p53 null cells. This augmentation of apoptosis was correlated with activated NF-kB and transcriptional upregulation of NOXA and p21 but not PUMA. This study however, did not demonstrate the transcriptional upregulation of NF-kB by p53. The authors concluded that the treatment of p53 proficient cancers with reovirus and Nutlin 3a would have a therapeutic advantage over treatment with reovirus alone. Since over 50% of cancer cells are p53 mutant the utilization of Nutlin3a in conjunction with reovirus therapy for all histologies in the clinic may be premature. Further, in the Pan et al. [43] study reovirus induction of PUMA assessed by real time PCR resulted only in 5–8 fold increases in p53 proficient or null HCT116 colorectal cells respectfully. In comparison, in the present investigation we see a 12–80 fold increase in PUMA expression in MCF7 and HTB133 cells respectively indicating that in BrCa, reovirus alone can induce substantial apoptosis via PUMA induction. Given the complex nature of the apoptotic signaling cascades that a cell undergoes after appropriate stimuli, the contribution by PUMA however, may not likely be sufficient to illicit complete apoptosis in these cells.

The reovirus sensitivity demonstrated in all 4 BrCa cell lines used in the present study correlated with activation of NF-kB as seen with qRT-PCR, westerns and EMSAs. Nuclear localized DNA bound NF-kB consisted of the p65/p50 complex indicative of canonical NF-kB signaling post reovirus treatment. Blocking NF-kB activity significantly reduced apoptosis confirming the proapoptotic nature of NF-kB in this system and supports the results of Conolly et al. [25] seen in Hela cells.

Apoptosis resulting from NF-κB signaling may also be due to the promoter activation of DRs and ligands such as Fas, Fas-L, TNF-α, and the TRAIL receptors DR4 and DR5 as predicted earlier [44, 45]. As shown in our microarray data upregulation of DR associated genes such as TNF-α induced protein 3, DR4, TNFR member6, TNF member1 and Fas associated factor were noted. Interestingly, in a previously reported gene array experiment involving HEK 293 cells and reovirus type 3 Abney failed to show alterations in expression of any of these DR associated genes or NF-kB related genes [46] despite the previous documentation of the importance of NF-kB activity in reovirus induced apoptosis in these cells [26]. The upregulation of the caspase associated genes and the BH3-only proapoptotic molecule PUMA seen in the present BrCa model suggest NF-kB to be an important mediator of cross talk between the extrinsic and the intrinsic apoptotic pathways and warrants further investigation. The contribution and functional involvement of individual DRs to apoptosis could only be confirmed following assessment of their downstream signaling post inhibitor blocking.

In an emerging era of personalized medicine, it is of utmost importance to predict early whether a tumour would be therapy sensitive or resistant. Although over 1000 patients have been treated with reovirus to date, no markers for reovirus sensitivity are yet available for such a prediction. It is only recently that evidence of PUMA induction by chemotherapeutic agents has emerged. As reported by Middleburg et al. [47], analysis of tissue biopsies from BrCa patients revealed PUMA mRNA induction by 6h post chemotherapy treatment. Elevated levels of PUMA and Bim expression were indicative of better prognosis in stage II and III colon cancer patients treated with 5-FU and shown to be an independent prognostic markers for disease free and overall survival [48]. Leukemic cells isolated from dexamethasone treated patients have shown elevated PUMA levels in treatment sensitive but not in treatment resistant patients [49] all indicating that PUMA is a good prognostic marker for therapeutic evaluation.

## Conclusions

This report presents the first line of evidence where NF-kB activates PUMA post reovirus therapy in BrCa and highlights the potential of using both molecules as predictive markers for reovirus sensitivity of BrCa. The extension of these findings to other histologies as predictive biomarkers is worthy of investigation in the future.

## Supporting Information

S1 Fig Effects of CAPE and ALLN on cell viability of HTB133 breast cancer cells. HTB 133 cells were grown in 96-well plates and treated with varying doses of CAPE and ALLN for 24 and 48 hours. Cell viability was assessed via the WST assay. (N = 3, ± SD).
(TIF)

S2 Fig Effects of CAPE and ALLN on cell viability of MCF7 breast cancer cells. MCF7 cells were grown in 96-well plates and treated with varying doses of CAPE and ALLN for 24 and 48 hours. Cell viability was assessed via the WST assay. (N = 3, ± SD).
(TIF)

S3 Fig Effects of CAPE and ALLN on reovirus progeny production. MCF7 and HTB 133 cells were grown in six-well plates and infected with either 40 MOI of reovirus or reovirus+CAPE (20μM) or reovirus+ALLN (10μM). Plates were incubated for varying time points up to 48 hours and frozen at -80C. Following three freeze-thaw cycles of the frozen cells, the supernatants were subjected to plaque titration on L929 cells. The experiment was repeated two times for each cell line.
(TIF)

S1 Table Gene validation data for real time PCR assays using the Taqman Human Endogenous Control plate. NV- No virus, DV Dead virus, LV–Live virus.
(DOCX)
