# Supplementary figures and images for: PUMA and NF-kB Are Cell Signaling Predictors of Reovirus Oncolysis of Breast Cancer
Source: PLoS One. 2017 Jan 18;12(1):e0168233. doi: 10.1371/journal.pone.0168233 (PMC5243128; doi:10.1371/journal.pone.0168233)

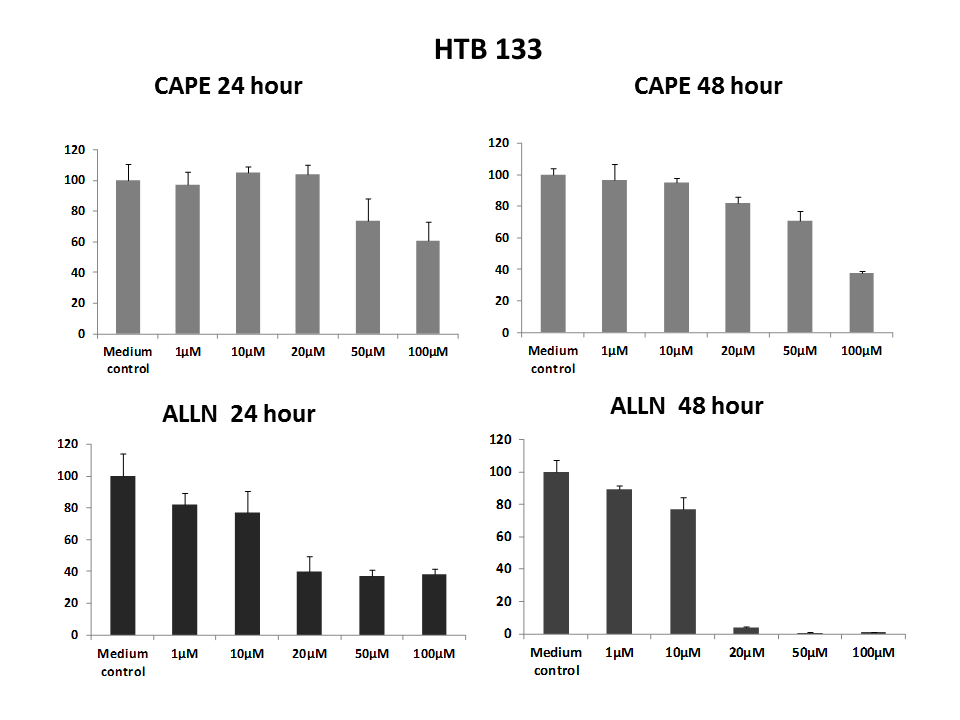

Supplement: S1 Fig — HTB 133 cells were grown in 96-well plates and treated with varying doses of CAPE and ALLN for 24 and 48 hours. Cell viability was assessed via the WST assay. (N = 3, ± SD). (TIF) [file pone.0168233.s001.tif]

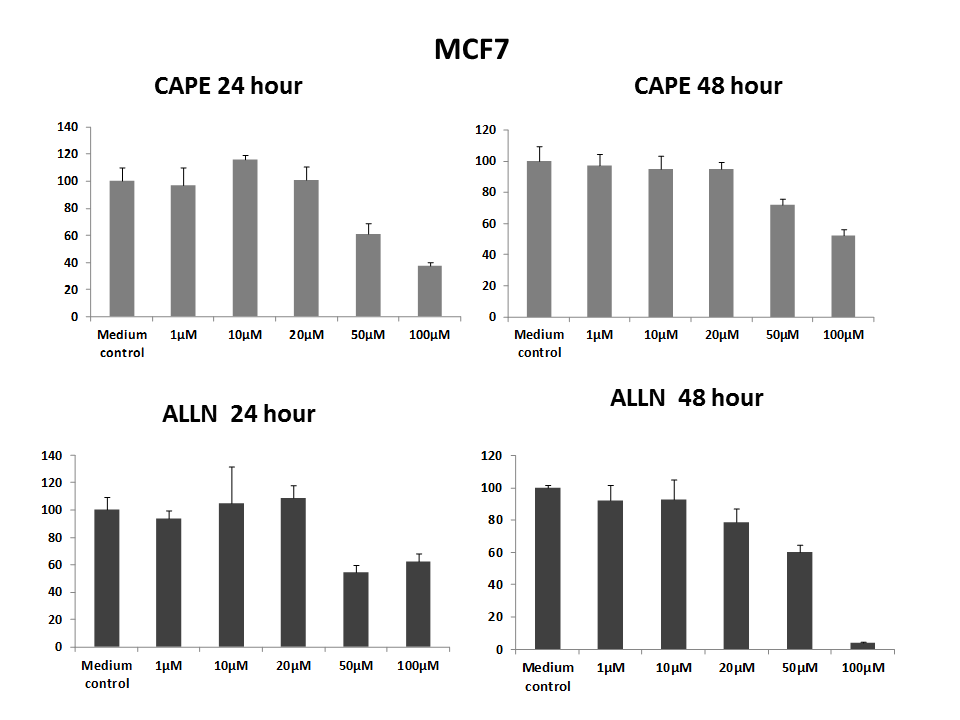

Supplement: S2 Fig — MCF7 cells were grown in 96-well plates and treated with varying doses of CAPE and ALLN for 24 and 48 hours. Cell viability was assessed via the WST assay. (N = 3, ± SD). (TIF) [file pone.0168233.s002.tif]

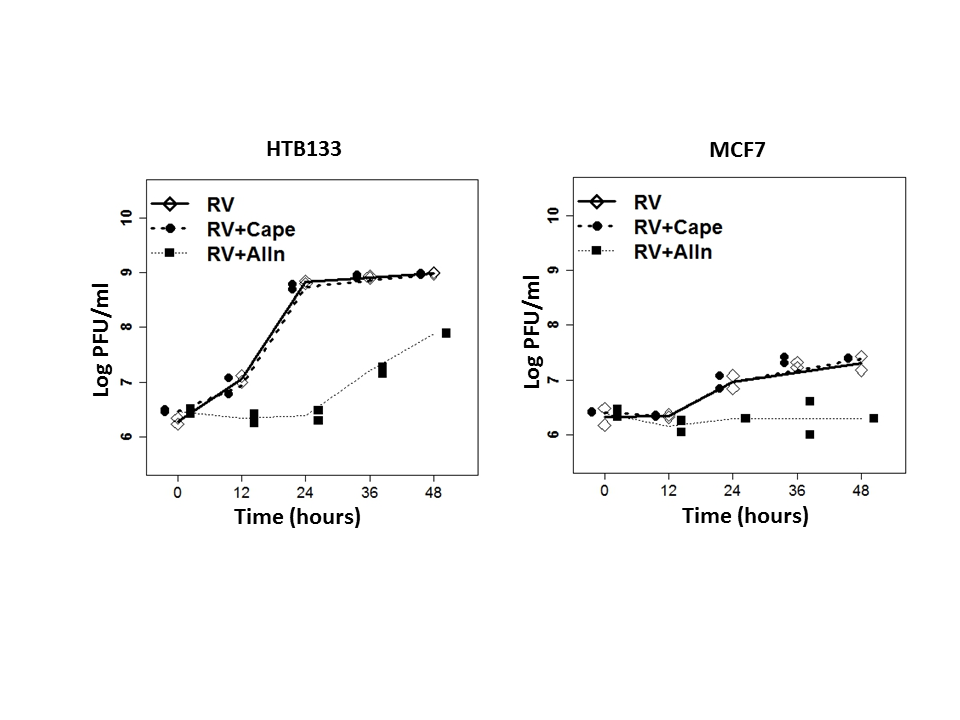

Supplement: S3 Fig — MCF7 and HTB 133 cells were grown in six-well plates and infected with either 40 MOI of reovirus or reovirus+CAPE (20μM) or reovirus+ALLN (10μM). Plates were incubated for varying time points up to 48 hours and frozen at -80C. Following three freeze-thaw cycles of the frozen cells, the supernatants were subjected to plaque titration on L929 cells. The experiment was repeated two times for each cell line. (TIF) [file pone.0168233.s003.tif]
