# Supplementary material for: PUMA and NF-kB Are Cell Signaling Predictors of Reovirus Oncolysis of Breast Cancer
Source: PLoS One. 2017 Jan 18;12(1):e0168233. doi: 10.1371/journal.pone.0168233 (PMC5243128; doi:10.1371/journal.pone.0168233)
Supplement: S1 Table — NV- No virus, DV Dead virus, LV–Live virus. (DOCX) [file pone.0168233.s004.docx]

**S1Table. Gene validation data for real time PCR assays using the Taqman Human Endogenous Control plate.** NV- No virus, DV Dead virus, LV – Live virus

| Sample Name | Target Name | Cт Mean | Sample Name | Target Name | Cт Mean | Sample Name | Target Name | **Cт Mean** |
| --- | --- | --- | --- | --- | --- | --- | --- | --- |
| MCF7 NV | IPC | **24.9204102** | MCF7 DV 48H | IPC | **25.0864315** | MCF7 LV 48H | IPC | **25.1042614** |
| MCF7 NV | 18S | **11.2258644** | MCF7 DV 48H | 18S | **10.9200354** | MCF7 LV 48H | 18S | **10.9400482** |
| MCF7 NV | huPO | **20.8271923** | MCF7 DV 48H | huPO | **20.909874** | MCF7 LV 48H | huPO | **22.3068848** |
| MCF7 NV | huBA | **19.2164097** | MCF7 DV 48H | huBA | **18.9234104** | MCF7 LV 48H | huBA | **19.7972698** |
| MCF7 NV | huCYC | **26.6633186** | MCF7 DV 48H | huCYC | **26.1733303** | MCF7 LV 48H | huCYC | **28.1032619** |
| MCF7 NV | huGAPDH | **19.2918205** | MCF7 DV 48H | huGAPDH | **19.0746822** | MCF7 LV 48H | huGAPDH | **20.3495464** |
| MCF7 NV | huPGK | **24.2498379** | MCF7 DV 48H | huPGK | **24.2937527** | MCF7 LV 48H | huPGK | **25.493515** |
| MCF7 NV | huB2m | **27.1415672** | MCF7 DV 48H | huB2m | **25.1249485** | MCF7 LV 48H | huB2m | **24.0359249** |
| MCF7 NV | huGUS | **26.9537792** | MCF7 DV 48H | huGUS | **26.6334629** | MCF7 LV 48H | huGUS | **27.806921** |
| MCF7 NV | huHPRT | **25.2968826** | MCF7 DV 48H | huHPRT | **25.3459225** | MCF7 LV 48H | huHPRT | **26.6987991** |
| MCF7 NV | huTBP | **27.8339539** | MCF7 DV 48H | huTBP | **27.6654644** | MCF7 LV 48H | huTBP | **29.0017605** |
| MCF7 NV | huTfR | **24.1577206** | MCF7 DV 48H | huTfR | **23.8575134** | MCF7 LV 48H | huTfR | **25.5010662** |
| HTB133 NV | IPC | **24.8083496** | HTB133 DV 48H | IPC | **24.7866764** | HTB133 LV 48H | IPC | **24.7521381** |
| HTB133 NV | 18S | **10.4965744** | HTB133 DV 48H | 18S | **10.6572905** | HTB133 LV 48H | 18S | **12.6272364** |
| HTB133 NV | huPO | **20.2236328** | HTB133 DV 48H | huPO | **21.0616264** | HTB133 LV 48H | huPO | **25.4531155** |
| HTB133 NV | huBA | **18.573101** | HTB133 DV 48H | huBA | **18.8259506** | HTB133 LV 48H | huBA | **23.5128708** |
| HTB133 NV | huCYC | **25.0978317** | HTB133 DV 48H | huCYC | **25.3036823** | HTB133 LV 48H | huCYC | **30.2602158** |
| HTB133 NV | huGAPDH | **19.2715969** | HTB133 DV 48H | huGAPDH | **19.1947803** | HTB133 LV 48H | huGAPDH | **24.647522** |
| HTB133 NV | huPGK | **23.934103** | HTB133 DV 48H | huPGK | **24.0799122** | HTB133 LV 48H | huPGK | **28.9793186** |
| HTB133 NV | huB2m | **24.3417721** | HTB133 DV 48H | huB2m | **21.7924671** | HTB133 LV 48H | huB2m | **23.8082943** |
| HTB133 NV | huGUS | **24.9969177** | HTB133 DV 48H | huGUS | **24.9007626** | HTB133 LV 48H | huGUS | **29.7923565** |
| HTB133 NV | huHPRT | **26.2114315** | HTB133 DV 48H | huHPRT | **26.5445709** | HTB133 LV 48H | huHPRT | **31.1906281** |
| HTB133 NV | huTBP | **27.1277065** | HTB133 DV 48H | huTBP | **27.3811512** | HTB133 LV 48H | huTBP | **31.8001785** |
| HTB133 NV | huTfR | **20.5556393** | HTB133 DV 48H | huTfR | **20.7442169** | HTB133 LV 48H | huTfR | **27.3390751** |
